# *KRAS*-mutant non-small cell lung cancer (NSCLC) therapy based on tepotinib and omeprazole combination

**DOI:** 10.1186/s12964-024-01667-x

**Published:** 2024-06-12

**Authors:** Rafael Rosell, Eloisa Jantus-Lewintre, Peng Cao, Xueting Cai, Baojuan Xing, Masaoki Ito, Jose Luis Gomez-Vazquez, Mireia Marco-Jordán, Silvia Calabuig-Fariñas, Andrés Felipe Cardona, Jordi Codony-Servat, Jessica Gonzalez, Kevin València-Clua, Andrés Aguilar, Carlos Pedraz-Valdunciel, Zahra Dantes, Anisha Jain, S Chandan, Miguel Angel Molina-Vila, Oscar Arrieta, Macarena Ferrero, Carlos Camps, Maria González-Cao

**Affiliations:** 1 Germans Trias i Pujol Research Institute, Badalona (IGTP), Barcelona, Spain; 2 IOR, Hospital Quiron-Dexeus Barcelona, Barcelona, Spain; 3 Laboratory of Molecular Biology, Germans Trias i Pujol Health Sciences Institute and Hospital (IGTP), Camí de les Escoles, s/n, 08916 Badalona, Barcelona, Spain; 4 Molecular Oncology Laboratory, Fundación Investigación Hospital General Universitario de Valencia, Valencia, Spain; 5 Trial Mixed Unit, Centro Investigación Príncipe Felipe-Fundación Investigación Hospital General Universitario de Valencia, Valencia, Spain; 6 Centro de Investigación Biomédica en Red de Cáncer, CIBERONC, Madrid, Spain; 7 Department of Biotechnology, Universitat Politècnica de València, Camí de Vera s/n, Valencia, 46022 Spain; 8 Joint Unit: Nanomedicine, Centro Investigación Príncipe Felipe-Universitat Politècnica de Valencia, Valencia, Spain; 9 Jiangsu Provincial Medical Innovation Center, Affiliated Hospital of Integrated Traditional Chinese and Western Medicine, Nanjing University of Chinese Medicine, Nanjing, China; 10 State Key Laboratory on Technologies for Chinese Medicine Pharmaceutical Process Control and Intelligent Manufacture, Nanjing University of Chinese Medicine, Nanjing, China; 11 The Quzhou Affiliated Hospital of Wenzhou Medical University, Quzhou Peoples Hospital, Quzhou, China; 12 Shandong Academy of Chinese Medicine, Jinan, China; 13 Department of Surgical Oncology, Research Institute for Radiation Biology and Medicine, Hiroshima University, Hiroshima, Japan; 14 Hospital Universitari de Bellvitge, Bellvitge Biomedical Research Institute (IDIBELL), L’Hospitalet de Llobregat, Barcelona, Spain; 15 Department of Pathology, Universitat de Valéncia, Valencia, Spain; 16 Institute of Research and Education, Luis Carlos Sarmiento Angulo Cancer Treatment and Research Center - CTIC, Bogotá, Colombia; 17 Pangaea Oncology, Hospital Quiron-Dexeus Barcelona, Barcelona, Spain; 18 Invitrocue, Barcelona, Spain; 19 Invitrocue, Munich, Germany; 20 Department of Microbiology, JSS Academy of Higher Education & Research, Mysuru, India; 21 National Institute of Cancerology (INCAN), Mexico City, Mexico; 22 Medical Oncology Department, General University Hospital of Valencia, Valencia, Spain

## Abstract

**Background:**

*KRAS-*mutant non-small cell lung cancer (NSCLC) shows a relatively low response rate to chemotherapy, immunotherapy and *KRAS*-G12C selective inhibitors, leading to short median progression-free survival, and overall survival. The *MET* receptor tyrosine kinase (c-*MET*), the cognate receptor of hepatocyte growth factor (HGF), was reported to be overexpressed in *KRAS*-mutant lung cancer cells leading to tumor-growth in anchorage-independent conditions.

**Methods:**

Cell viability assay and synergy analysis were carried out in native, sotorasib and trametinib-resistant *KRAS*-mutant NSCLC cell lines. Colony formation assays and Western blot analysis were also performed. RNA isolation from tumors of *KRAS*-mutant NSCLC patients was performed and *KRAS* and *MET* mRNA expression was determined by real-time RT-qPCR. In vivo studies were conducted in NSCLC (NCI-H358) cell-derived tumor xenograft model.

**Results:**

Our research has shown promising activity of omeprazole, a V-ATPase-driven proton pump inhibitor with potential anti-cancer properties, in combination with the MET inhibitor tepotinib in *KRAS*-mutant G12C and non-G12C NSCLC cell lines, as well as in G12C inhibitor (AMG510, sotorasib) and MEK inhibitor (trametinib)-resistant cell lines. Moreover, in a xenograft mouse model, combination of omeprazole plus tepotinib caused tumor growth regression. We observed that the combination of these two drugs downregulates phosphorylation of the glycolytic enzyme enolase 1 (ENO1) and the low-density lipoprotein receptor-related protein (LRP) 5/6 in the H358 *KRAS* G12C cell line, but not in the H358 sotorasib resistant, indicating that the effect of the combination could be independent of ENO1. In addition, we examined the probability of recurrence-free survival and overall survival in 40 early lung adenocarcinoma patients with *KRAS* G12C mutation stratified by *KRAS* and *MET* mRNA levels. Significant differences were observed in recurrence-free survival according to high levels of *KRAS* mRNA expression. Hazard ratio (HR) of recurrence-free survival was 7.291 (*p* = 0.014) for high levels of *KRAS* mRNA expression and 3.742 (*p* = 0.052) for high *MET* mRNA expression.

**Conclusions:**

We posit that the combination of the V-ATPase inhibitor omeprazole plus tepotinib warrants further assessment in *KRAS*-mutant G12C and non G12C cell lines, including those resistant to the covalent *KRAS* G12C inhibitors.

**Supplementary Information:**

The online version contains supplementary material available at 10.1186/s12964-024-01667-x.

## Background

Kirsten rat sarcoma viral oncogene homologue (*KRAS*) mutations are relevant drivers in non-small-cell lung cancer (NSCLC), particularly in lung adenocarcinoma, with higher frequency occurring in Caucasian patients compared to Hispanic and Asian patients [1–3]. In a retrospective analysis of 1,194 patients diagnosed with *KRAS*-mutant NSCLC, the presence of *KRAS* G12C mutations (glycine-to-cysteine substitution at codon 12) was identified in 46% of the patients. In contrast, *KRAS* non-G12C mutations were observed in the remaining 54% [4]. When considering treatment modalities such as chemotherapy and/or immunotherapy, the median survival durations from the time of diagnosis were similar, 13.1 and 13.4 months, respectively. In patients treated with platinum-based chemotherapy as first- or second-line, progression-free survival (PFS) was meager, 3 versus 2.8 months [4]. Of note, *KRAS* co-occurring *STK11/LKB1* alterations and KEAP1 mutations have been identified as a major cause of primary resistance to anti-PD-1 or anti-PD-L1 immune checkpoint inhibitors in *KRAS*-mutant NSCLC [5–7].

Sotorasib (AMG510) is a small molecule that selectively inhibits *KRAS* G12C. The Food and Drug Administration (FDA) granted accelerated approval to sotorasib for the treatment of NSCLC patients with *KRAS* G12C mutations who had received at least one previous line of therapy [8]. Among heavily pretreated patients with *KRAS* G12C NSCLC from the phase 1 study of the CodeBreak 100 trial, a response rate of 37.1% was observed. In the phase 2 study, 33.9% of patients also showed a partial response, with median PFS of 6.3 and 6.8 months, respectively [9, 10]. The median survival was 12.5 months in the phase 2 study and treatment-related grade 3 adverse events were observed in 19.8% of patients, consisting of diarrhea, nausea, fatigue, and arthralgia, and an increase in aspartate and alanine aminotransferase levels were observed in 19.8% of patients [10]. Adagrasib (MRTX849) is another potent, oral, irreversible, and selective *KRAS* G12C inhibitor that has demonstrated clinical activity in *KRAS* G12C-mutant NSCLC patients (KRYSTAL-1 trial) previously treated with chemotherapy and immunotherapy, with a response rate of 42.9%, a median PFS of 6.5 months, and a median overall survival (OS) of 12.6 months. Treatment-related grade 3 events were seen in 42.8% of patients [11]. At the recommended phase 2 dose (600 mg twice a day) the most common treatment-related adverse events were nausea, diarrhea, vomiting and fatigue [12]. Mechanisms of resistance to sotorasib and adagrasib include a plethora of additional mutations in *KRAS* (G12V/D/R or G13D, Y96C, and others), HRAS, NRAS, and BRAF, as well as loss-of-function mutations in NF1 and PTEN, oncogenic fusions in ALK, RET, BRAF, RAF1 and FGFR3, EGFR alterations, *MET* amplification, and histologic transformation to squamous-cell carcinoma [13–15].

Poor clinical outcomes in *KRAS*-mutant NSCLC patients are attributed to the limitations of monotherapy with *KRAS* G12C inhibitors, which rapidly becomes insufficient due to the emergence of collateral targetable pathways known as ‘collateral dependencies.’ These dependencies exhibited synergistic responses with EGFR inhibitors and, additionally, with inhibitors of FGFR, SHP2, PI3K, or CDK4/6 [16–20]. While various mechanisms could regulate *MET* amplification, both *KRAS*-mutant G12C and non-*KRAS*-mutant G12C NSCLCs may share a dependence on *MET* and, as a result, could benefit from MET inhibition therapy. *MET* dependency in NSCLC stems from the seminal report where the expression of *c-MET* was found in all the NSCLC tissues examined (*n* = 23) and most (89%) of the cell lines (*n* = 9) [21]. A specific MET inhibitor, SU11274, was effective in eight of the nine cell lines tested, with IC_50’s_ ranging from 0.8 to 4.4 µmol/L. Notably, SU11274 inhibited MET/HGF activity in both A549 and H1993 cells, even when subjected to stimulation with HGF (40 ng/mL, 7.5 min) [21]. Following the initial observation that targeting *MET* could offer therapeutic value in NSCLC treatment, subsequent investigations unveiled the essential role of enhanced *MET* expression and signaling in facilitating anchorage-independent growth within *KRAS*-mutant cancer cells. Consequently, MET inhibitors could be of benefit to *KRAS*-mutant tumor patients [22]. Mitogen-activated protein kinase (MEK) inhibitor, selumetinib, was combined with docetaxel versus docetaxel alone in previously treated *KRAS*-mutant NSCLC patients without improving PFS in front of docetaxel alone [23]. Intriguingly, it was observed that selumetinib diminishes inhibitory phosphorylation of *MET* at serine 985 with potential enhancement of HGF and EGF-induced AKT phosphorylation in *KRA*S-mutant NSCLC cell lines [24].

It was noted that pretreatment with omeprazole increased the cytoplasmic retention of cytotoxic drugs. Specifically, omeprazole was found to sensitize cancer cell lines to cisplatin, which was associated with the inhibition of V-H+-ATPase (V-ATPase) activity, resulting in elevated pH levels within lysosomes [25]. Moreover, inhibition of V-ATPase with concanamycin A inhibited the internalization of c-*MET* protein induced by HGF exposure in adult T-cell leukemia/lymphoma cell lines [26] suggesting that the combination of V-ATPase inhibitor plus MET inhibitor could be synergistic. Omeprazole 80 mg orally twice daily for 4–7 days was administered before neoadjuvant chemotherapy and continued until surgery in patients with operable triple-negative breast cancer. The addition of omeprazole to neoadjuvant chemotherapy produces a higher pathologic complete response rate [27]. Herein, we have conducted a study using *KRAS* G12C sotorasib-resistant cell lines, *KRAS* non-G12C trametinib (MEK inhibitor)-resistant cell lines, as well as parental cell lines, to investigate the potential of a therapeutic strategy involving the pre-treatment with omeprazole in combination with tepotinib, a selective MET inhibitor, and actinomycin D, a transcriptional inhibitor of ribosomal DNA genes that activates TP53 [28]. Actinomycin D inhibited *KRAS* mRNA and *KRAS* protein expression induced by a *KRAS* G12C tool compound inhibitor, ARS-1620, in mutant *KRAS* G12C lung cancer cells [29].

## Methods

### Cells and reagents

The human NSCLC NCI-H358 (*KRAS* G12C), NCI-H23 (*KRAS* G12C), H1792 (*KRAS* G12C) A549 (*KRAS* G12S) and H460 (*KRAS* Q61H) cell lines were purchased from the ATCC. The human drug-resistant cancer cell lines H358SR and H23SR resistant to AMG510 (sotorasib) and A549TR and H460TR resistant to trametinib were kindly provided by the General University Hospital Research Foundation of Valencia as well as the PC435 (*KRAS* G12C) cell line derived from a pleural effusion of a lung adenocarcinoma patient (Supplementary Table 1). The resistant H358SR, H23SR, A549TR, and H460TR cells were produced by exposing parental cells to increasing sub lethal concentrations of the drugs. All cell lines were authenticated by short term tandem repeat method (AmpFSTR Idntifiler Plus PCR Amplification kit) and routinely verified as mycoplasma free. The cells were maintained in a humidified atmosphere of 5% CO2 at 37ºC in RPMI-1640 medium (Sigma-Aldrich Corp.) supplemented with 10% FBS, 1% penicillin–streptomycin and 1% amphotericin B.

AMG510 (#HY-114,277) was obtained from MedChemExpress (Monmouth Junction); tepotinib (#S7067), and omeprazole (#S1389) were purchased from Selleck Chemicals. Actinomycin D (#11,805,017) was purchased from Thermo Fischer Scientific. All drugs were aliquoted and stored at − 80ºC until use. Tepotinib was dissolved in dimethyl sulfoxide to a final concentration of 10 mM and warmed up to 50ºC in a water bath. All other compounds were dissolved in dimethyl sulfoxide to a final concentration of 10 mM (omeprazole, actinomycin D, and AMG510).

### In vitro growth inhibition assay

To analyze the potential antitumor effects of combining tepotinib with omeprazole, we employed the human lung adenocarcinoma cell lines, H358 and A459, along with their resistant counterparts: H358SR (resistant to sotorasib) and A549TR (resistant to trametinib). These resistant cell lines were generated by stepwise long-term exposure to sotorasib or trametinib.

Cells were seeded into 96-well round-bottom plates (2000 cells/well), as previously described [30]. After 24 h of incubation with supplemented RPMI, cells were either treated with omeprazole, tepotinib, or their combination. For the later, cells were supplemented with tepotinib upon 24 h of omeprazole pre-treatment. After 72 h of incubation, cell viability was assessed using the MTT reagent (tetrazolium-based semiautomated colorimetric 3 (4,5-dimethylthiazol-2-yl)-2,5-diphenyltetrazolium bromide, Sigma) following the manufacturer’s instructions. Formazan crystals in viable cells were solubilized in 100 µl DMSO and spectrophotometrically quantified using a microplate reader (Infinite M Plus) at 550 nm of absorbance. Colorimetric values were expressed as a percentage of that observed in untreated cells. The concentration that gives half-maximal response (EC_50_ ) of each drug was then calculated using Combenefit software.

One-way analysis of variance (ANOVA) applying Prism 10.0 (Graphpad, San Diego, CA, USA) was utilized for evaluating the differences among multiple groups. Statistics results with p value below 0.05 were considered statistically significant.

To assess the combined effects of the drugs, we employed the Combenefit software (https//sourceforge.net/projects/combenefit/). This software employs a dose-matrix approach to predict potential synergy, antagonism, or additivity between drugs. It utilizes different models, including the Bliss independence model, the Loewe additivity model, and the Highest Single Agent (HSA) model [31]. The Bliss model is considered appropriate to assess drug effects with independent responses with distinct modes of action. The HAS model states that the expected combination is equal to the higher effect of individual compounds. The study was carried out in accordance with the principles of the Declaration of Helsinki and its later amendments, under an approved protocol of the Institutional Review Board of the Hiroshima University.

### Colony formation assay

Cells were plated in six-well plates at 500 cells/well in RPMI, 10% FBS, as previously described [30]. Cells were cultured for 24 h, and the media was then replaced with RPMI, 1% FBS with or without drugs. After 72 h, the media was removed and replaced with fresh media without drugs for a total of 10 days. At the end of the experiment, the media was removed, and cells were washed with phosphate-buffered saline (PBS). Colonies were fixed and stained simultaneously with 0.5% crystal violet in 10% ethanol for 15 min. The stain was aspirated, and the wells were washed with deionized water until the background was clear.

### Western blotting

Cells were seeded in 10% FBS-supplemented RPMI and, the next day, cells were treated with the drug alone or in combination at a concentration of 10µM (tepotinib), 100µM (omeprazole) or the corresponding IC50 (sotorasib). Cells were collected and centrifuged, and pellets were washed with cold PBS and re-suspended in ice-cold radio-immunoprecipitation assay buffer (50mM Tris- hydrochloric acid in pH 7.4, 1% Nonidet P-40, 0.5% sodium deoxycholate, 0.1% sodium dodecyl sulfate [SDS], 150mM sodium chloride, 1mM ethylenediaminetetraacetic acid, 1mM sodium vanadate and 50mM sodium fluoride) containing protease inhibitor mixture. Then, cell lysis was performed by sonication and followed by centrifugation at 14,000 rpm for 15 min at 4 °C. Resulting supernatant was collected as the total cell lysate. Next, lysates containing 30 µg proteins were electrophoresed on 10% SDS-polyacrylamide gels (Life Technologies) and transferred to polyvinylidene difluoride membranes (BIO-RAD laboratories). Membranes were blocked in Odyssey blocking buffer (LI-COR Biosciences). All target proteins were immunoblotted with appropriate primary and either IRDye-conjugated or horseradish peroxidase-conjugated secondary antibodies. Chemiluminescent bands (HRP-conjugated) were detected in a ChemiDoc MP Imaging System (Bio-Rad). Proteins were probed using primary antibodies (1:1000 dilution) specific for E-cadherin (#3195, Cell Signaling), enolase-1 (#3810, Cell Signaling), *MET* (#4560, Cell Signaling), Phospho-*MET* (Tyr1234/1235) (D26) (#3077, Cell Signaling), phospho-*MET* (Tyr1003) (13D11) (#3135, Cell Signaling), phospho-Akt (Ser473) (#9271, Cell Signaling), phospho-p44/42 MAPK (Erk1/2) (Thr202/Tyr204) (#9101, Cell Signaling), non-phospho (active) β-catenin (Ser33/37/Thr41)(D13A1)(#8814, Cell Signaling), phospho-LRP6/5 (Ser1490)(#2568, Cell Signaling), RGS3 (#ab2564, abcam), and secondary antibodies (1:10000) IRDye® 800CW Goat anti-Mouse IgG (926-32210, LI-COR) and IRDye® 800CW Goat anti-Rabbit IgG (926-32211). β-actin was used as an internal control to confirm equal gel loading.

### In vivo experiments

Nu/Nu mice (20 ± 2 g) were obtained from Changzhou Cavens Lab Animal Company (Changzhou, Jiangsu, China). All animals were maintained in a clean facility in Jiangsu Province Academy of Traditional Chinese Medicine (Nanjing, Jiangsu, China). Mice were kept in IVC cages (5 per cage) with free access to food and water, at 20 °C and 50 ± 20% relative humidity under a 12:12 h light/dark cycle and pathogen free conditions. All procedures were based on the Guide for Care and Use of Laboratory Animals of the National Institutes of Health and approved by the Institutional Animal Care and Use Committee of Jiangsu Province Academy of Traditional Chinese Medicine (SYXK 2021-0025). A suspension of 4 × 106 H358 cells resuspended in PBS mixed 1:1 with basement membrane extract were injected subcutaneously into the right flank of the mice. When established tumors reached a palpable size (~ 100 mm3), the mice were randomized into the following groups for a three-week treatment period: vehicle group (4% DMSO + 40% PEG300 + 5% Tween-80 + 51% saline), tepotinib alone group (5 mg/kg), omeprazole alone group (75 mg/kg), actinomycin D alone group (40 µg/kg), tepotinib plus omeprazole group, and tepotinib plus omeprazole plus actinomycin D group. The body weight and tumor size were measured once every two days. Tumor volume was calculated as follows: length*(width2)*0.5. At the end of the experiments, all animals were sacrificed, and the tumors were excised and weighed.

### Quantitative real-time polymerase chain reaction (RT-PCR) analyses

RNA was converted into Template complementary DNA (cDNA) by using the M-MLV (Moloney Murine Leukemia Virus Reverse Transcriptase) retro-transcriptase enzyme. cDNA was added to the Taqman Universal Master Mix (Applied Biosystems) in a 12.5 µl reaction with specific primers and probes for the *KRAS* and *MET* genes. Quantification of gene expression was performed using the ABI Prism 7900HT Sequence Detection System (Applied Biosystems) and was calculated according to the comparative Ct method. Final results were determined as follows: 2^−(ΔCt sample−ΔCt calibrator),^ where ΔCt values of the calibrator and sample are determined by subtracting the Ct value of the target gene from the value of the endogenous gene (β-actin). Commercial RNA controls were used as calibrators (Liver and Lung; Stratagene, La Jolla, CA, USA). In all quantitative experiments, a sample was considered not evaluable when the standard deviation of the Ct values was > 0.30 in two independent analyses. The following primers were used:

*KRAS*, purchased from Thermo Fisher Scientific, Assay ID: Hs00364282_m1, Catalog num: 4331182; MET, Forward Primer: 5’ TCACCATAGCTAATCTTGGGACATC 3’, Reverse Primer: 5’ GTTGATGGTCCTGATCGAGAAAC 3’, Probe: 5’ TCGCTTCATGCAGGTTG 3’; β -actin, Forward Primer: 5’ TGAGCGCGGCTACAGCTT 3’, Reverse Primer: 5’, TCCTTAATGTCACGCACGATTT 3’, Probe: 5´ACCACCACGGCCGAGCGG 3’.

The impact of *KRAS* mRNA expression on first progression (FP) and overall survival (OS) were validated using Kaplan-Meier plotter (kmplot.com), which includes data from GEO, EGA, and TCGA. FP and OS curves were created in M0 lung cancer cases using “Affy ID 204010_s_at”, “auto selected best cutoff”, and “JetSet best probe set” setting.

## Results

### Omeprazole potentiates the antitumor effect of tepotinib in *KRAS*-mutant lung cancer cell lines

**Fig. 1:**
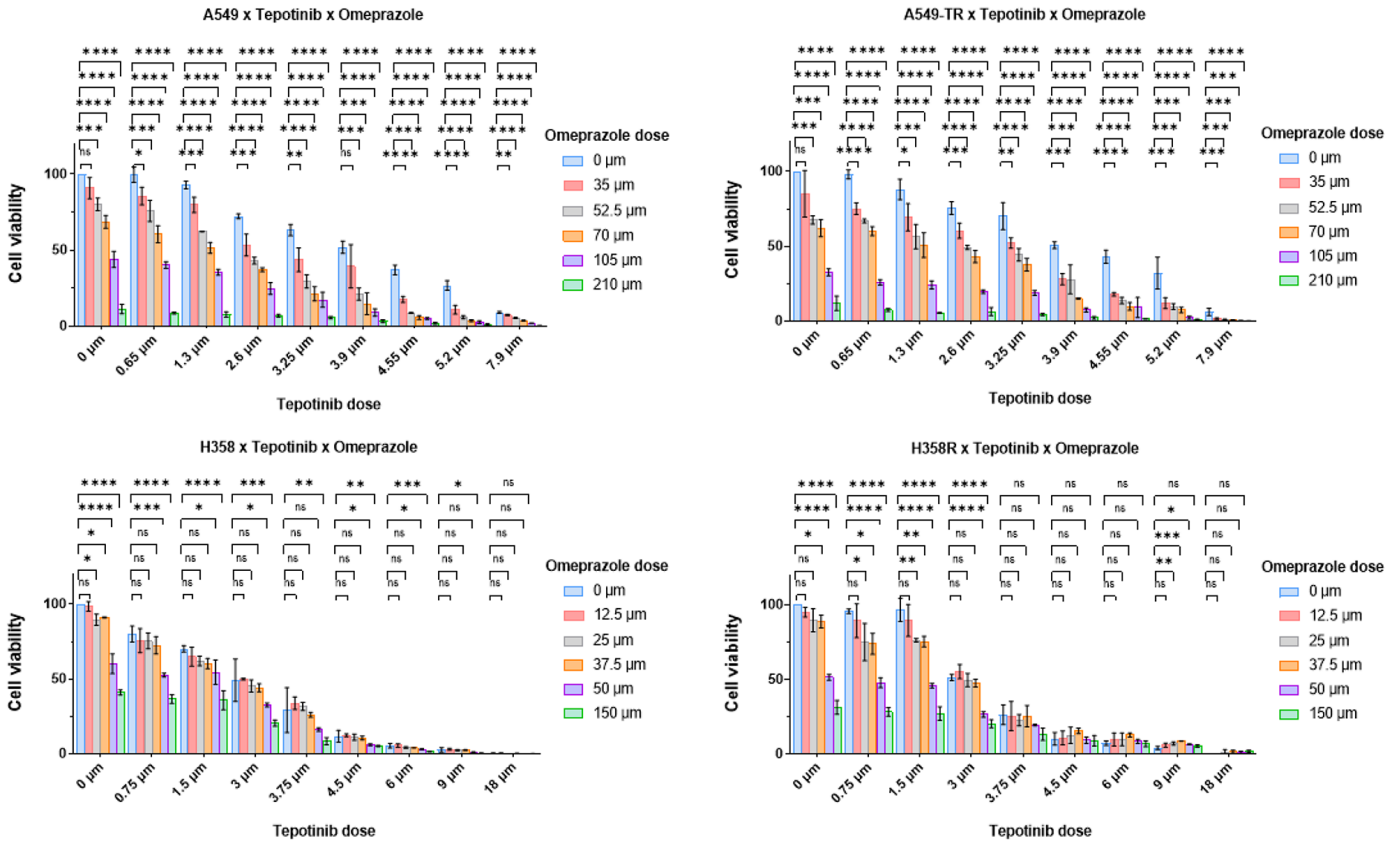
Combination of tepotinib plus omeprazole potentiates cell viability inhibition. Lung cancer cells H358 and A549 and the resistant cell lines to sotorasib (H358R) or trametinib (A549TR) were exposed to increasing concentrations of tepotinib combined with increased concentrations of omeprazole for 72 h. Cell viability was analyzed by MTT assay. The experiments were performed in triplicate. The tepotinib dose was as follows: 0, 0.75, 1.5, 3, 3.75, 4.5, 6, 9, 18 (µM) for H358 and H358R cell lines, and 0, 0.65, 2.6, 3.25, 3.9, 4.55, 5.2, and 7.9 for A549 and A549TR cell lines. The omeprazole dose range was: 0, 12.5, 25, 37.5, 50 and 150 (µM) for H358 and H358R cells and 0, 35, 52, 70, 105 and 210 (µM) for A549 and A549TR cells. Graphs show the mean +/- SD of 3 independent replicates. One-way ANOVA test was used, ⁎*p* < 0.05, ⁎⁎*p* < 0.01, ⁎⁎⁎*p* < 0,0001, ns = not significant

To analyze the potential antitumor effects of combining tepotinib with omeprazole, we employed the *KRAS*-mutant NSCLC cell lines, H358 and A459, and their derived resistant clones H358SR (sotorasib-resistant) and A549 TR (trametinib-resistant). These resistant cell lines were generated by stepwise long-term exposure to sotorasib or trametinib. The cell lines were treated for 72 h with tepotinib, omeprazole or the combination. Cell viability in the A549 and A549TR cell lines progressively decreased as the cells were treated with increasing doses of tepotinib (range 0-7.9 µM) and omeprazole (range 0-150 µM). The Analysis of Variance (ANOVA) test was employed to analyze the differences among the means and to determine the statistical significance of the interaction between tepotinib and omeprazole. Most of the dose combinations between the two drugs showed statistical significance in both the parental and resistant cell lines. Cell viability in the H358 and H358R cell lines progressively decreased as the cells were treated with increasing doses of teponitib (range 0–18 µM) and omeprazole (range 0-150 µM). Using the ANOVA test we observed that the best combination results were with the lower dose of tepotinib (0.75, 1.5 and 3 µM) and the higher dose of omeprazole (50 and 150 µM). We concluded that the combination of tepotinib with several doses of omeprazole resulted in reduced cell viability by MTT. Thus, the combination notably enhanced the effectiveness of reducing cell viability (Fig. 1 and Supplementary Fig. 1).

**Fig. 2:**
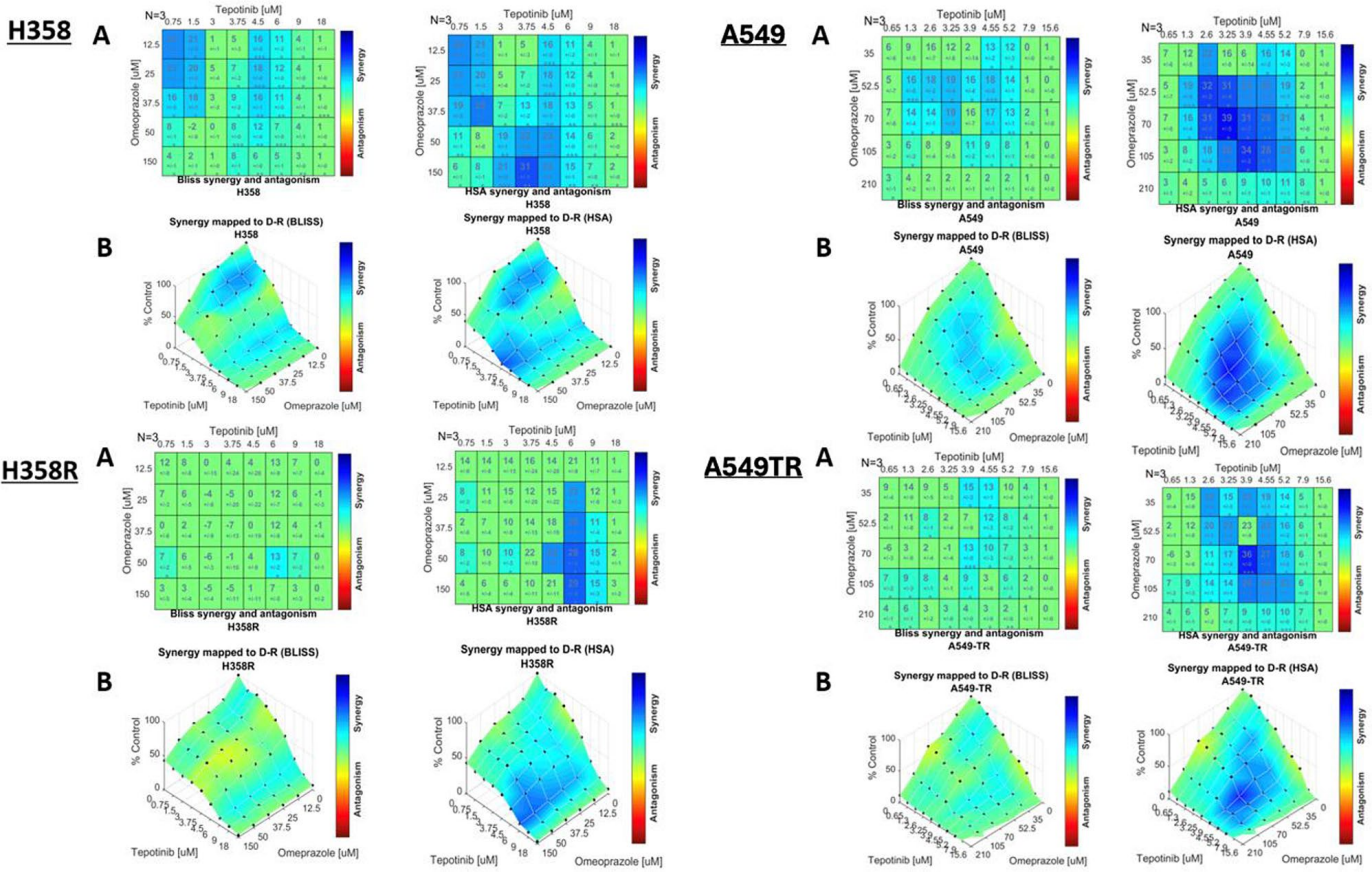
Isobologram analysis and synergism determination: Dose-response matrix is depicted showing tepotinib and omeprazole dual drug assays in NSCLC cells (H358, A549) and resistant cell lines (H358R, A459TR) using Combenefit Software (Bliss & HSA Models). This matrix portrays the interaction between eight doses of tepotinib and five doses of omeprazole across four distinct cell lines: H358, A549 and their resistant derivatives, H358SR and A549TR. **(A)** Two-drug combination dose response surface expressed as a percentage of the control value. The plot portrays the efficacy of each of the dual-drug combinations. **(B)** Synergy scores, shown in matrix format, calculated according to the Bliss and HSA methods. The blue boxes indicate synergy between tepotinib and omeprazole, while the green boxes represent additivity, and the yellow, orange, or red boxes signify antagonism. The number of biological replicates (N) is indicated at the top left of the matrix. The number below the synergy score is the standard deviation

Isobologram analysis and synergism determination was performed across all the cell lines. The EC50 of tepotinib, calculated with the Combenefit software, in A549, A549TR, H358 and H358R cell lines were respectively, 3.68, 3.93, 3.06 and 4,93 µM. The EC50 of omeprazole, in A549, A549TR, H358 and H358R were respectively, 94.1, 75.6, 58.2 and 105 µM. In the H358 and A549 cell lines, low and intermediate doses of tepotinib showed synergy over the entire dose range of omeprazole. In the H358R sotorasib-resistant cell line, synergism was confined at the 6 µM dose of tepotinib analyzed with the HSA method, meanwhile the effects were additive when the Bliss method is used. In the trametinib-resistant cell line, A549TR showed synergy with low and intermediate doses of tepotinib with most of the omeprazole doses. The H358R cellular model exhibits less synergism between tepotinib and omeprazole than the other three cellular models. Antagonism was not observed in any drug dose combination (Fig. 2).

**Fig. 3:**
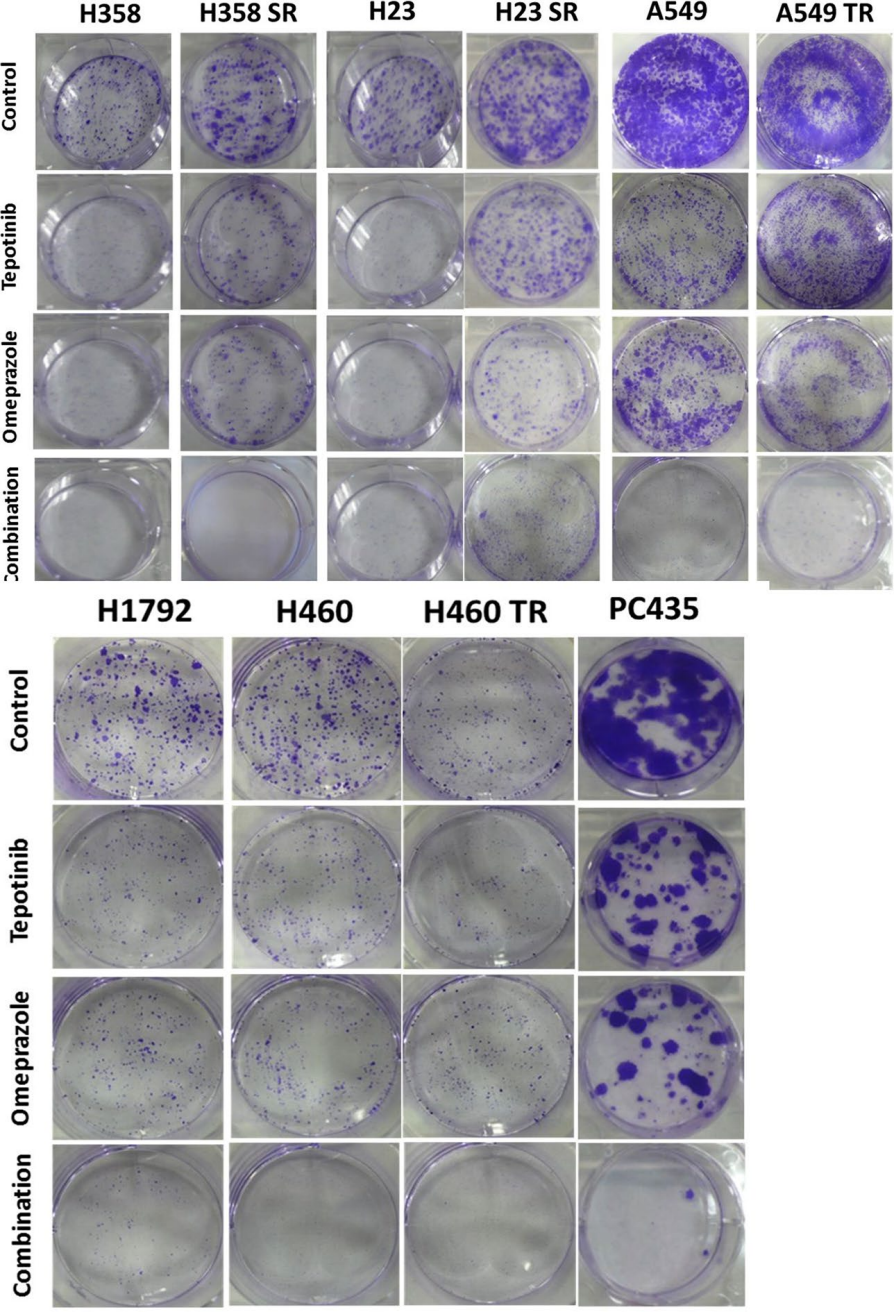
Clonogenic formation assay upon treatment of *KRAS*-mutant cell lines grown in monolayer cultures with tepotinib, omeprazole or the double combination. The cells were grown in six-well plates (500 cells/well) for 24 h and then left untreated or treated with tepotinib, omeprazole and the double combination. After 72 h, media was replaced with fresh media without drugs. After seven more days cells were washed and stained with crystal violet and then photographed. Images are representative of at least three independent experiments

In line with previous results, the colony formation assays demonstrated enhanced efficacy of combining omeprazole and tepotinib in multiple *KRAS* G12C-mutant lung cancer cell lines (H358, H32, H1792 and PC435) and *KRAS* non-G12C-mutant cell lines (A549 and H460). Representative wells of clonogenic growth and inhibition are depicted in Fig. 3. The antiproliferative effect was also observed in the sotorasib resistant cells (H358SR and H23SR), and in trametinib (MEK inhibitor) resistant cell lines (A549TR and H460TR). The effect of Actinomycin D, alone or in combination with tepotinib and omeprazole, was observed in a colony formation assay (Supplementary Fig. 2).

### Western blot analysis of the omeprazole and tepotinib combination in H358 and H358 sotorasib-resistant *KRAS*-mutant lung cancer cell lines

**Fig. 4:**
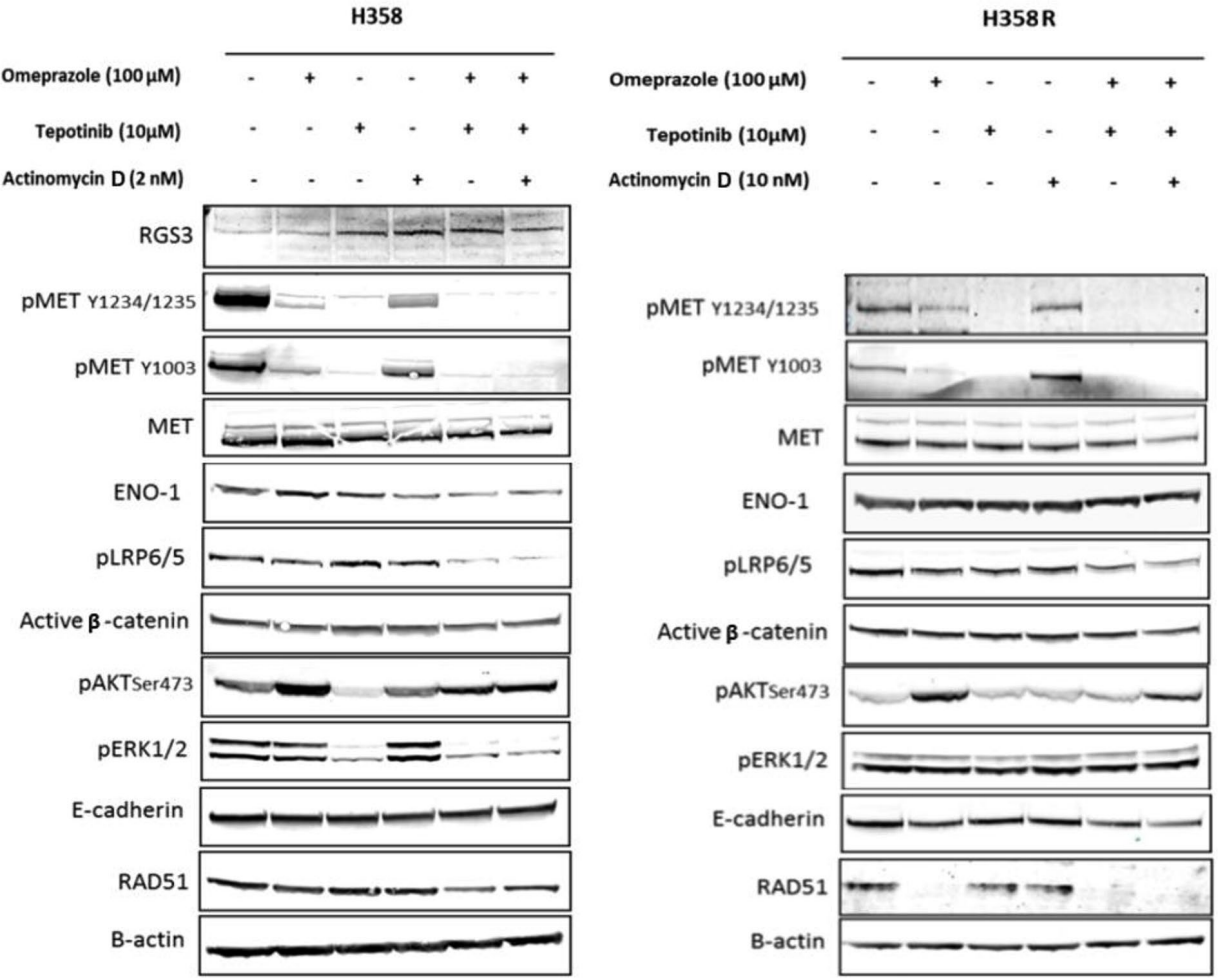
Effects of single treatments or tepotinib plus omeprazole combination with or without actinomycin D on proteins involved in the downstream signaling of the *Wnt*, *MET* and *KRAS* pathways. Western blot analysis showed protein levels in parental H358 and H358R cells treated with the single drugs (omeprazole 100 µM, tepotinib 10 µM, actinomycin 2µM) or the combinations. Protein extracts were taken at 24 h after treatment. β-actin was used as loading control. Data were generated from a minimum of three replicates

To study the mechanism through which omeprazole mediated synergism with tepotinib, we considered the role of V-ATPase inhibitors. Western blot analysis was carried out in the H358 cell line. It was previously reported that expression levels of regulator of G protein signaling 3 (RGS3), a GTPase-activating protein, was a readout of GDP-bound *KRAS* state, predicting sensitivity to *KRAS* inhibitors [32]. A higher RGS3 expression was associated with lower mutant *KRAS* output in lung cancers harboring G12C or any *KRAS* mutation. Moreover, RGS3^−/−^ cells had an attenuated response to G12C inhibitors compared with their isogenic RGS3 WT cells [32]. Therefore, we gauge RGS3 expression as a readout of *KRAS*-GTP level inhibition. *MET* is the target of tepotinib and its phosphorylation in tyrosines 1234/1235 and 1003 was inhibited with tepotinib, omeprazole, and actinomycin at the drug doses tested. The combination of these drugs increased the inhibition of *MET* phosphorylation. Tepotinib increased the expression of RGS3 and decreased the phosphorylation of AKT and Erk1/2. The combination of tepotinib plus omeprazole decreased the phosphorylation of LRP5/6. We indeed see that in the H358 cell line the combination of omeprazole and tepotinib with or without actinomycin D increases RGS3 levels while reducing ENO1, LRP5/6, ERK1/2 phosphorylation, as well as baseline *MET* phosphorylation at Y1234/1235 (Fig. 4). In the H358 sotorasib-resistant cell line (H358R), there is no effect on ENO-1, pAkt, and pErk1/2 protein levels with either of the combinations of omeprazole plus tepotinib, with or without actinomycin D. There are no significant changes in active β-catenin and E-cadherin.

### In vivo antitumor efficacy of tepotinib in combination with omeprazole and actinomycin D

**Fig. 5:**
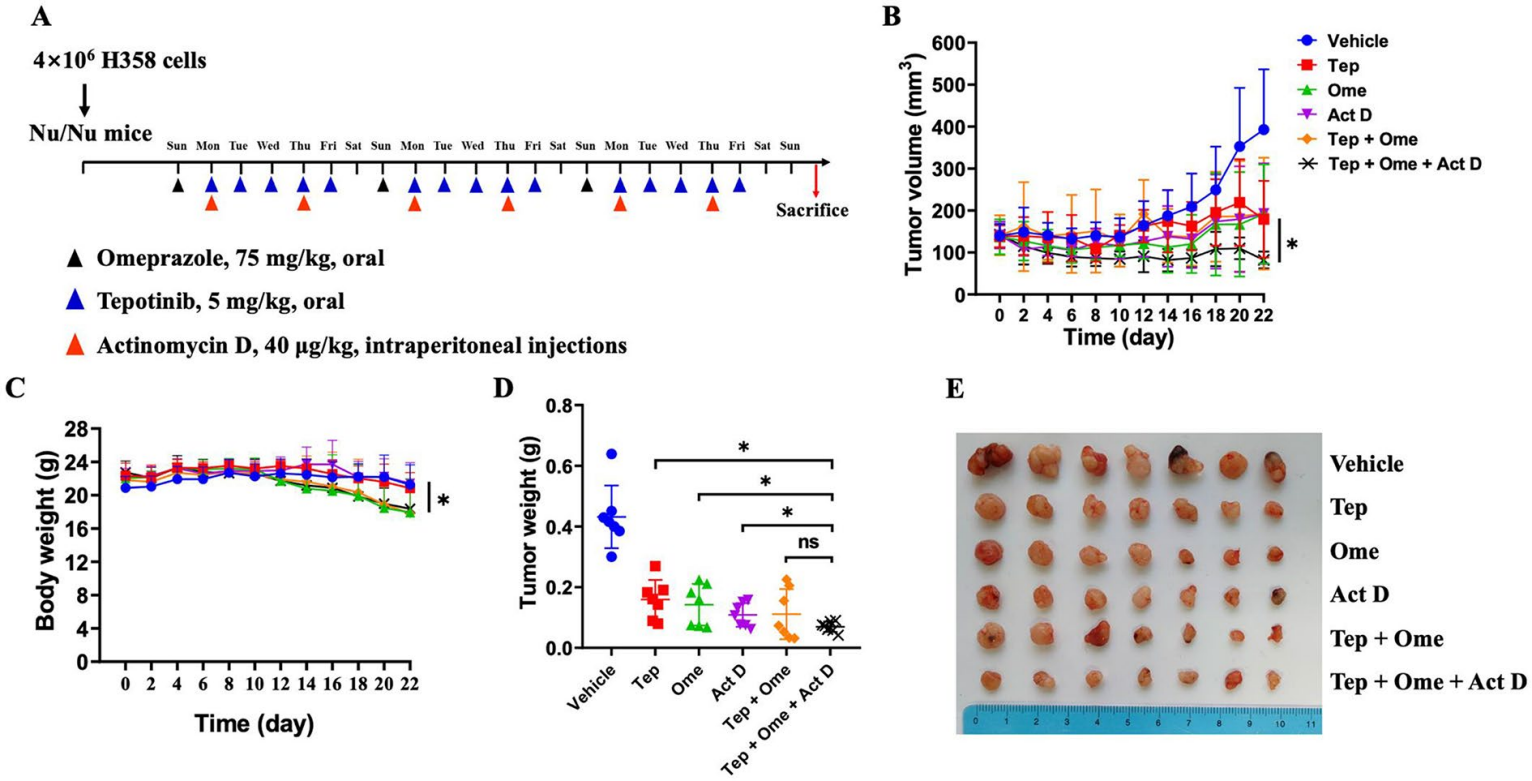
In vivo antitumor efficacy of tepotinib in combination with omeprazole and actinomycin D. **A**. Time schedule for the H358 cells subcutaneous implantation and drug treatment groups, mice were randomly grouped into 6 groups: vehicle group, tepotinib group, omeprazole group, actinomycin D group, tepotinib + omeprazole group, tepotinib + omeprazole + actinomycin D group. **(B)** Tumor volumes for subcutaneous H358 tumor. **(C)** Mice body weight in each group. **(D)** Subcutaneous tumor weight in each group. **(E)** Representative H358 tumor pictures. (*n* = 7). Data were analyzed using unpaired t-test comparisons. * *P* < 0.05, compared to the tepotinib + omeprazole + actinomycin D group

We next evaluated the in vivo antitumor efficacy of tepotinib in combination with omeprazole and actinomycin D. In the H358 *KRAS*-mutant lung cancer xenograft animal model, a three-week treatment with tepotinib was administered, both with and without omeprazole pretreatment, as well as with or without actinomycin D (Fig. 5A). The results indicated a more pronounced tumor regression in the group that received the triple combination of tepotinib, omeprazole, and actinomycin D (*p* = 0.0150), in contrast to the groups treated with tepotinib alone (*p* = 0.0485) or omeprazole alone (*p* = 0.0482) (Fig. 5B, D, and E). Stronger tumor regression was observed with tepotinib plus omeprazole plus actinomycin D (*p* = 0.0150, versus tepotinib group; *p* = 0.0485, versus omeprazole group; *p* = 0.0482 versus actinomycin D group) (Fig. 5D). While the tumor size in the group receiving tepotinib in combination with omeprazole and actinomycin D was smaller compared to the group receiving only tepotinib and omeprazole, statistically, there was no significance (Fig. 5D, E). There was also no significant difference between the tepotinib plus omeprazole group and the single drug group (tepotinib plus omeprazole versus tepotinib, *p* = 0.8041; tepotinib plus omeprazole versus omeprazole, *p* = 0.9889; tepotinib plus omeprazole versus actinomycin D, *p* = 0.9988) (Fig. 5D, E). Three groups of animals containing omeprazole experienced weight loss, possibly due to gastrointestinal side effects of omeprazole (Fig. 5C).

### Recurrence-free survival and overall survival in early lung adenocarcinoma patients with *KRAS* G12C mutation stratified by *KRAS* and *MET* mRNA expression

**Fig. 6:**
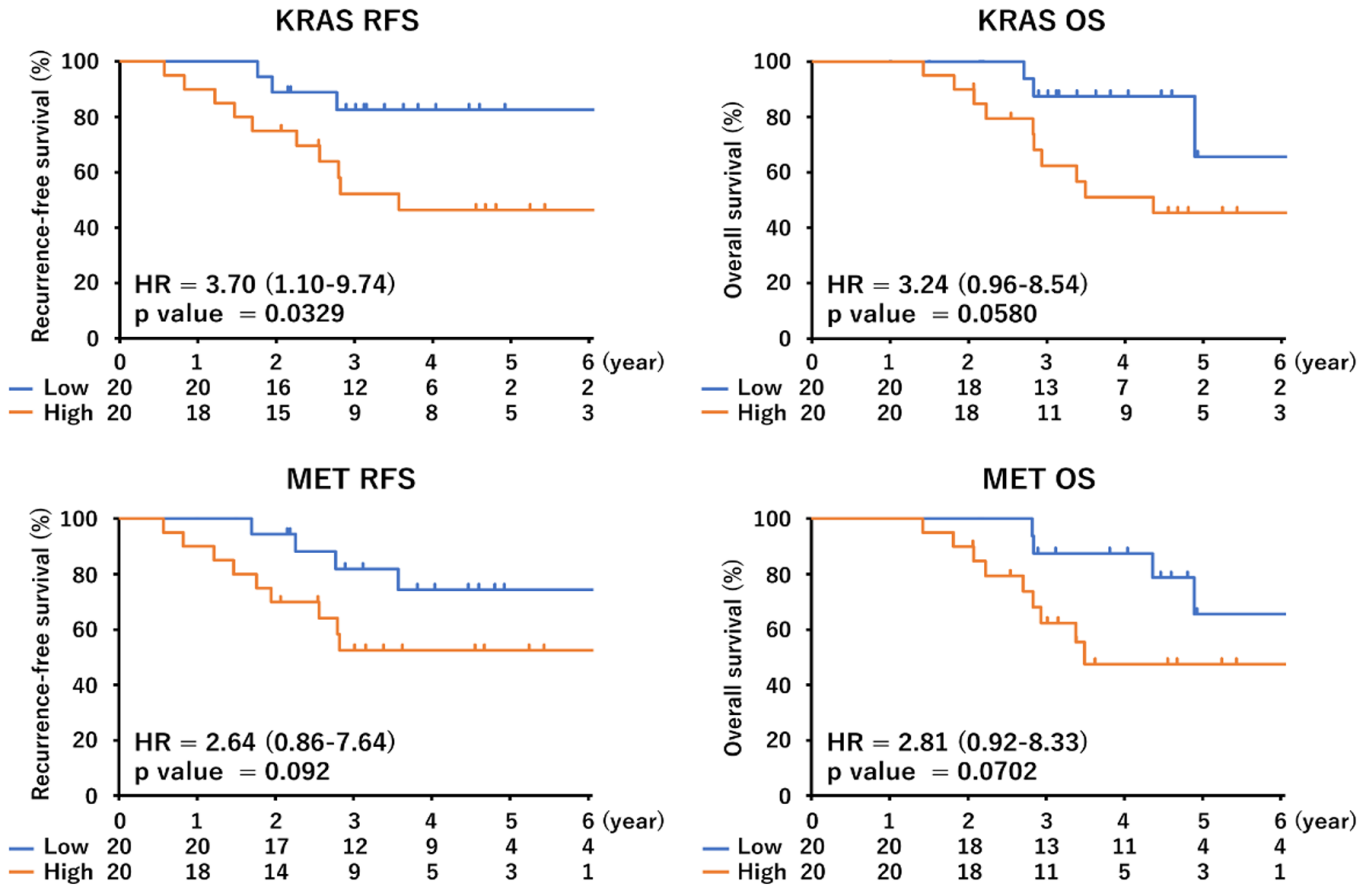
Recurrence-free survival and overall survival curves based on the mRNA expression level of *KRAS* or *MET*. Kaplan-Meier curves were created by dividing patients with the median of mRNA expression

We analyzed a cohort of surgically resected tumor tissues of 40 *KRAS* G12C mutation-positive lung adenocarcinoma patients for *KRAS* and *MET* mRNA expression by quantitative PCR assays. Gene expression levels were dichotomized using the median as cut-off. The clinicopathological characteristics of the 40 patients are shown in Table 1. Kaplan-Meier graphs representing the probability of RFS and OS stratified to *KRAS* and *MET* mRNA expression are shown in Fig. 6. Significant differences were observed in RFS according to *KRAS* mRNA expression (hazard ratio [HR] for recurrence, 3.70; *p* = 0.0329; HR of 7.291; *p* = 0.014 in the multivariate analysis, Table 2). A week positive correlation was observed between *KRAS* and *ME*T mRNA levels (*r* = 0.22, *p* > 0.05). Although there was a trend towards a higher risk of recurrence with elevated *MET* mRNA expression, the HR observed for these patients was not statistically significant (Fig. 6; Table 2). The HR for OS according to *KRAS* high expression was 3.257 (*p* = 0.073) in the univariate analysis and 4.261 (*p* = 0.038) in the multivariate analysis (Table 3). The HR for high *MET* expression was 3.742 (*p* = 0.052) in the multivariate analysis (Table 3). The mRNA expression of *KRAS* was examined by TCGA data analysis. The cohort with high KRAS expression showed a trend towards worse progression-free survival, and overall survival was significantly worse in this cohort (Supplementary Fig. 2).

**Table 1:** Clinicopathological features of the 40 reviewed cases

Clinicopathological characteristics	Patients (*N* = 40)
**Age, years**	
Median (range, interquartile range)	69.5 (32–86, 63–74)
**Sex, N (%)**	
Male	23 (57.5)
Female	17 (42.5)
**Pathological invasive size, mm**	
Median (range, interquartile range)	18 (2–54, 10–34.5)
**Histological subtype, N (%)**	
MIA	5 (12.5)
Lepidic predominance	8 (20.0)
Papillary predominance	18 (45.0)
Acinar predominance	0 (0)
Micropapillary predominance	2 (5.0)
Solid predominance	6 (15.0)
Invasive adenocarcinoma (NOS)	1 (2.5)
**IASLC histological grade**	
MIA	5 (12.5)
G1	8 (20.0)
G2	12 (30.0)
G3	14 (35.0)
Unknown	1 (2.5)
**Pleural invasion, N (%)**	
PL 0	33 (82.5)
PL 1	7 (17.5)
**Intrapulmonary metastasis, N (%)**	
PM 0	35 (87.5)
PM 1	5 (12.5)
**Post-operative platinum doublet + ICI**	
Received	4 (10.0)
**Post-recurrence platinum doublet + ICI**	
Received	3 (7.5)

*Abbreviations* AIS, adenocarcinoma in situ; IASLC, International Association for the Study of Lung Cancer; ICI, immune checkpoint inhibitor; MIA, minimally invasive adenocarcinoma; NOS, not otherwise specific; PI, pleural invasion; PM, pulmonary metastasis

**Table 2:** Uni- and multivariate analyses for recurrence free survival (RFS) according to *KRAS* or *MET* mRNA expression (*N* = 40) recurrence-free survival

	Univariate analysis		Multivariate analysis	
Variable	HR (95% CI)	*P-*value	HR (95% CI)	*P-*value
Age	1.018 (0.965–1.075)	0.514	1.040 (0.979–1.103)	0.202
Sex (Male)	1.839 (0.566–5.976)	0.311	1.079 (0.176–6.625)	0.935
Pathological invasive size (mm)	1.046 (1.008–1.086)	0.018	1.083 (1.018–1.152)	0.012
IASLC Grade (Grade 2, 3)	1.240 (0.372–4.131)	0.727	0.083 (0.007–0.984)	0.049
Pleural invasion (Positive)	4.096 (1.334–12.58)	0.014	0.487 (0.049–4.884)	0.541
Intrapulmonary metastasis (Positive)	3.246 (0.881–11.96)	0.077	8.102 (1.351–48.59)	0.022
Post-operative platinum doublet + ICI (Received)	3.424 (0.937–12.51)	0.063	0.924 (0.121–7.081)	0.940
KRAS expression (High)	3.708 (1.019–13.49)	0.047	7.291 (1.351–35.32)	0.014
MET expression (High)	2.653 (0.815–8.642)	0.105	2.295 (0.618–8.522)	0.215

**Table 3:** Uni- and multivariate analyses for overall survival (OS) according to *KRAS* or *MET* mRNA expression (*N* = 40). overall survival

	Univariate analysis		Multivariate analysis	
Variable	HR (95% CI)	*P-*value	HR (95% CI)	*P-*value
**Age**	1.726 (0.530–5.619)	0.365	1.071 (1.002–1.144)	0.044
**Sex (Male)**	1.017 (0.967–1.069)	0.518	0.948 (0.151–5.931)	0.954
**Pathological invasive size (mm)**	4.018 (1.308–12.35)	0.15	1.117 (1.039–1.201)	0.003
**IASLC Grade** **(Grade 2, 3)**	1.278 (0.384–4.252)	0.690	0.036 (0.002–0.654)	0.018
**Pleural invasion (Positive)**	4.018 (1.308–12.35)	0.015	0.623 (0.740–5.25)	0.663
**Intrapulmonary metastasis (Positive)**	2.742 (0.750–10.02)	0.127	4.849 (0.733–32.09)	0.102
**Post-recurrence platinum doublet + ICI (Received)**	3.296 (0.889–12.21)	0.074	7.447 (0.468–118.5)	0.155
**KRAS expression (High)**	3.257 (0.894–11.86)	0.073	4.261 (1.080–16.81)	0.038
**MET expression (High)**	2.856 (0.872–9.351)	0.083	3.742 (0.990–14.14)	0.052

*Abbreviations* CI, confidence interval; HR, hazard ratio; IASLC, International Association for the Study of Lung Cancer, ICI, Immune checkpoint inhibitor

## Discussion

Our preclinical findings pointed out that MET inhibition could be essential as a therapeutic approach for both *KRAS* G12C subtype and non-G12C subtype, regardless of resistance to previous treatment prior lines and of co-mutational patterns. Ma and colleagues identified that *MET* is overexpressed and activated in NSCLC cell lines and tumor tissues [21]. Expression of *MET* was found in all (100%) of the NSCLC tissues examined and most (89%) of the cell lines. 61% of tumor tissues strongly expressed total *MET*, especially adenocarcinomas (67%). Specific expressions of phospho-*MET* (Y1003) and Y1230/1234/1235 were seen by immunohistochemistry. *MET* expression was preferentially observed at the NSCLC tumor invasive fronts. SU11274 (a MET inhibitor) abrogated cell viability in *MET* expressing NSCLC cells. It also inhibited hepatocyte growth factor (HGF)-induced phosphorylation of *MET* [21]. *MET* knockdown using small interfering RNA restores sensitivity to sotorasib in H23 (*KRAS* G12C) resistant cells. *MET* activation reinforced *RAS* cycling from its inactive form to its active form. Crizotinib (MET inhibitor) restored sensitivity to sotorasib. Dual inhibition led to tumor shrinkage in sotorasib-resistant xenograft mice [33]. The G12C inhibition treatment induced *KRAS* mRNA and *KRAS* protein expression, which is manifested as soon as 24 to 72 h after starting treatment in most *KRAS* G12C cell lines. Inhibiting new *KRAS* synthesis with the transcription inhibitor actinomycin D or *KRAS*-specific siRNA prevented *KRAS*-GTP rebound during the G12C inhibition treatment [29]. A noticeable effect of actinomycin D was the increased phosphorylation of AMP-activated protein kinase (AMPK) phosphorylation in acute myeloid leukemia cell lines [28]. It is tempting to posit that actinomycin D may restore *LKB1* function in *KRAS*-mutant cell lines harboring *LKB1* mutations. Further investigation is warranted to explore this potential connection.

We hypothesize that V-ATPase inhibitors obstruct the activation of *Wnt* signaling by inhibiting the plasma membrane receptor Frizzled when it forms a complex with the low-density lipoprotein receptor-related protein (LRP5/6) [34]. Conversely, the activation of LRP5/6 phosphorylation, driven by HGF, promotes canonical *Wnt* signaling [35]. Recent evidence also suggests that *ENO1* is associated with *MET*, activating the *Wnt* coreceptor and inducing epithelial-to-mesenchymal transition in lung cancer [36]. This leads us to theorize that in *KRAS*-mutant lung tumors, HGF, acting through ENO1, activates *MET* downstream effectors and *Wnt* signaling through the Frizzled coreceptor LRP5/6.

The results indicate that the combination of omeprazole plus tepotinib plays a central role in *KRAS*-mutant NSCLC with G12C subtype or non-G12C subtype, either sensitive or resistant to sotorasib or trametinib, respectively (Fig. 3). Mechanistically the combination of omeprazole and tepotinib downregulates the signaling pathway of MET-ENO1-LRP5/6, as well as upregulates RGS3 in the parental cell line. Our findings in the H358 xenograft model also confirm the tumor growth inhibition of omeprazole plus tepotinib with or without actinomycin D addition.

The current study examined the preclinical use of the MET inhibitor, tepotinib, a type Ib MET inhibitors, similar to capmatinib and savolitinib. Notwithstanding, the use of other MET inhibitors such as crizotinib (type Ia inhibitor) [37] could shed further light on the potential utility of MET inhibitors in the treatment of *KRAS*-mutant NSCLC, encompassing both *KRAS* G12C and non-G12C *KRA*S-mutant substitutions. An important issue is that pan-*RAS* or multi-*RAS* inhibitors [38] do not inhibit *NRAS* or *HRAS*, which, although they may avoid potential toxicities, cannot prevent feedback reactivation of the *RAS-MAPK* pathway through wild-type *NRAS* and *HRAS*, as reported with selective *KRAS* G12C inhibitors [39]. Elevated levels of *NRAS* cooperate with the oncogenic activity of *KRAS* G12D in pancreatic ductal adenocarcinoma [40]. It could be of further interest to pursue preclinical studies examining the effect of MET inhibitors compared to pan-RAS inhibitors in determining whether the efficacy of MET inhibitors can circumvent the activation of wild-type RAS [41, 42]. Studies with sotorasib or the pan-RAS inhibitor RMC-6236 have shown induction of *MAP2K4-JNK-Jun* signaling through inhibition of DUSP4, leading to increased expression of *ERBB2* and *ERBB3* [43]. Of interest is the fact that combining a pan-*RAS* inhibitor with a mutant-selective *KRAS* G12C inhibitor has been proposed to avoid or attenuate any reactivation of wild-type *RAS* proteins [44]. Therefore, it is tempting to posit that MET inhibitors could display some differences with pan-*RAS* inhibitors and allele-specific *KRAS* G12C inhibitors to avoid the activation of wild-type RAS isoforms or other signaling pathways. Moreover, it is currently unknown what effect MET inhibition may have in preventing *MRA*S activation, which has been observed through Scribble destabilization and YAP activation following treatment with sotorasib and adagrasib in *KRAS* G12C mutant cell lines [45]. Mechanistically, further research should be pursued to understand the potential activation of YAP, as well as the trimeric holoenzyme MRAS: SHOC2:PP1C as a potential mechanism of resistance [46]. Additionally, the roles of HUWE1 and valosin-containing protein, which act as regulators of MRAS: SHOC2:PP1C complex stability, should be investigated [47].

Clinical trials assessing the combination of *KRAS* G12C inhibitors with SHP2 are ongoing. The approach to inhibit SHP2, a key non-receptor protein tyrosine kinase that serves as an upstream platform in the activation of growth factor receptors, is very appealing [38]. However, in colorectal cancer cell lines harboring the *KRAS* G12C mutation, the combination of MRTX1133 with EGFR blockade has shown greater synergy than other combinations, including SHP2 inhibition [41]. In the present study, we looked for the combination of tepotinib as a *MET* inhibitor and omeprazole for its possible interception of the LRP5/6 *Wnt* co-receptors following *MET* activation via enolase1 and HGF, as previously reported [36]. Enolase, a glycolytic metalloenzyme, catalyzes the conversion of 2-phosphoglycerate to phosphoenolpyruvate and plays an important role in glycolysis. It is also interconnected with other metabolic pathways, such as fatty acid synthesis. It has been identified that the *KRAS* mutation induces specific induction of fatty acid synthase (FASN) and cerulenin, a specific inhibitor of FASN, decreasing the proliferation of *KRAS*-mutant lung adenocarcinoma cell lines, A549 and H1299) [48]. Omeprazole and other proton pump inhibitors have been shown to reduce homologous DNA damage repair, thereby augmenting the effect of radiotherapy and certain cytotoxic drugs [49]. Likewise, as stated in the Introduction, omeprazole was shown to inhibit FASN and improve the efficacy of neoadjuvant chemotherapy in breast cancer patients [27]. A FASN inhibitor, TVB-3664, has shown synergistic effects with MRTX849 (adagrasib) in lung adenocarcinoma cells with *KRAS* G12C and loss of heterozygosity for any RAS, including an in vivo xenograft model [50]. Therefore, these findings suggest the importance of exploring combinations of *KRAS* G12C inhibitors with FASN inhibitors, including omeprazole. Particularly relevant is the consideration of the loss of heterozygosity in *KRAS*-mutant cells. The selection of the *KRAS* G12C inhibitor could be of particular interest since it has been described that sotorasib displays an extensive covalent binding to over 300 off-target proteins, including KEAP1 [51]. As commented at the onset of the article, co-mutations of *LKB1* have a negative impact on overall survival in *KRAS*-mutant lung cancer patients receiving immune checkpoint inhibitors [5, 6], and *LKB1* deficiency decreases the HLA-A, HLA-B and HLA-C mRNA expression that integrates MHC-I [32]. It has been shown that FASN inhibition with orlistat or TVB-2640 can enhance MHC-I levels and improve the response to immune checkpoint inhibitors in hepatocellular carcinoma [52]. *KRAS*-mutant cell lines (both G12C and non-G12C) with *LKB1* mutations or deletions exhibit a lack of STING [53]. As a matter of interest, *MET* induces phosphorylation of UPF1 and promotes a decrease in *STING* mRNA levels [54]. This circumstantial evidence suggests that MET inhibition in the H23 *KRAS*-mutant cell line with *LKB1* mutation warrants further preclinical research. Furthermore, *LKB1* has been shown to regulate Scribble-YAP signaling [55]. Our results offer some insights, but there is still insufficient evidence to conclude that MET inhibitors have broad activity in *KRAS*-mutant cell lines. Many questions remain unanswered, including the potential synergism with selective *KRAS* G12C inhibitors and the need to further clarify the synergism with omeprazole or other agents identified as FASN inhibitors. Moreover, the addition of actinomycin needs to be investigated more thoroughly. Actinomycin D has been shown to restore promyelocytic leukemia (PML) nuclear bodies in acute myeloid leukemia [28]. Additionally, PML in breast cancer cells has been correlated with fatty acid oxidation [56]. It has been reported that actinomycin D, in combination with an angiotensin receptor antagonist that increases drug permeability, inhibits *Wnt* signaling in *KRAS* lung cancer models, including mouse Lewis lung carcinoma 1 and the human A549 cell line [57]. Enhanced activity was demonstrated by combining a selective *KRAS* G12C inhibitor, AMG510, with carboplatin [58]. However, cisplatin or carboplatin have not been explicitly analyzed in clinical trials of combinations with *KRAS* G12C inhibitors and chemotherapy [38]. Nevertheless, it is plausible that glycolysis and fatty acid oxidation could lead to cisplatin or carboplatin resistance, as has been described in *KRAS*-mutant cell lines and ovarian cancer cell lines [59]. It is tempting to posit that FASN inhibition, by accumulating malonyl-CoA, could neutralize carnitine palmitoyltransferase 1 A (CPT1A), the rate-limiting enzyme of fatty acid β-oxidation [60].

Our study does not aspire to do more than highlight the potential interest of MET inhibition. We acknowledge that MET inhibition cannot replace the significant advancements attained with selective *KRAS* inhibitors as well as with pan-*RAS* inhibitors. Certainly, the findings of the study are inconclusive. We can infer that omeprazole activity, as seen in the mice model, could be multifactorial. It is tempting to speculate that the inhibition of FASN could be relevant. Therefore, in light of recent evidence, it could potentially re-sensitize cells to carboplatin or cisplatin, as well as immune checkpoint inhibitors.

Although *MET* signaling could be relevant in *KRAS*-mutant NSCLC patients regardless of the type of *KRAS* codon 12 mutations (G12C or non-G12C), further studies are warranted. Establishing patient-derived organoid protocols in lung cancer patients can permit screening of omeprazole combined with tepotinib or other drugs in three-dimensional organoids to validate the potential clinical efficacy and utility of MET inhibitors with omeprazole or other V-ATPase inhibitors in the management of *KRAS*-mutant NSCLC. Furthermore, the assessment of *KRAS* and *MET* mRNA levels could also be of interest to be examined as a potential predictive biomarker.

## Conclusions

We conclude from the study carried out that further preclinical *MET*-oriented research should be pursued, and the observations of the study presented could pave the way for further actions in the field of *KRAS*-mutant lung adenocarcinoma.

### Electronic supplementary material

Below is the link to the electronic supplementary material.

Supplementary Material 1

## Data Availability

No datasets were generated or analysed during the current study.

## Abbreviations

AKT: Protein Kinase B
ALK: Anaplastic Lymphoma Kinase
BRAF: v-raf murine sarcoma viral oncogene homolog B1
CDK4: Cyclin-Dependent Kinase 4
CDK6: Cyclin-Dependent Kinase 6
c-MET: Mesenchymal-Epithelial Transition Factor
ENO1: Enolase 1
ERK1/2: Extracellular signal-regulated kinase 1 and 2
FGFR: Fibroblast Growth Factor Receptor
G12C: Glycine-to-Cysteine Substitution At Codon 12
HGF: Hepatocyte Growth Factor
KEAP1: Kelch-like ECH-Associated Protein 1
KRAS: Kirsten Rat Sarcoma Viral Oncogene Homologue
LKB1: Liver Kinase B1
LRP5: Low-density Lipoprotein Receptor-Related Protein 5
LRP6: Low-density Lipoprotein Receptor-Related Protein 6
MET: Mesenchymal Epithelial Transition
MRAS: Muscle RAS Oncogene Homolog
NRAS: Neuroblastoma ras viral oncogene homolog
OS: Overall Survival
PD-L1: Programmed Death-Ligand 1
PI3K: Phosphatidylinositol-3-kinase
PFS: Progression-Free Survival
RAF1: Rapidly Accelerated Fibrosarcoma
RET: Rearranged During Transfection
SHP2: Src Homology 2
STK11: Serine/Threonine Kinase 11
V-ATPase: Vacuolar-type ATPase
