# Supplementary material for: KRAS-mutant non-small cell lung cancer (NSCLC) therapy based on tepotinib and omeprazole combination
Source: Cell Commun Signal. 2024 Jun 12;22:324. doi: 10.1186/s12964-024-01667-x (PMC11167791; doi:10.1186/s12964-024-01667-x)
Supplement: Supplementary file 1 — Supplementary Material 1 [file 12964_2024_1667_MOESM1_ESM.docx]

**Supplementary Table 1**. Mutational status of *KRAS*, *TP53*, *STK11*, *KEAP* and *PIK3CA* in lung cancer cell lines

| **Cell line** | **Gender** | **Age** | **Histology** | **Gene mutational status** | | | | |
| --- | --- | --- | --- | --- | --- | --- | --- | --- |
|  |  |  |  | **KRAS** | **TP53** | **KEAP** | **STK11** | **PIK3CA** |
| **PC435 (*)** | Male | 70 | ADC | c.34G>T p.(Gly12Cys) |  | NA |  | c.3140 A>G p.(His1047Arg) |
| **H358** | Male | unspecified | ADC | c.34G>T p.(Gly12Cys) | gene deletion |  |  |  |
| **H23** | Male | 51 | ADC | c.34G>T p.(Gly12Cys) | c.738G>C p.(Met246Ile) |  | c.996G>A p.(Trp332Ter) |  |
| **H460** | Male | unspecified | LCC | c.183A>T p.(Gln61His) |  | c.? p.(Asp236His) | c.109C>T p.(Gln37Ter) | c.1633G>A p.(Glu545Lys) |
| **A549** | Male | 58 | ADC | c.34G>A p.(Gly12Ser) |  | c.? p.(Gly333Cys) | c.109C>T p.(Gln37Ter) |  |
| **H1792** | Male | 50 | ADC | c.34G>T p.(Gly12Cys) | c.672+1G>A | c. 1549T>G p.(Gly462Trp) |  |  |

Data obtained from ATCC, Expàsy cellosaurus Database, Blanco et al (61) and Goldstein et al [2].

(*) Data from NGS performed by Oncomine Comprehensive Assay Plus . NA: not analyzed. (61) Blanco R, Iwakawa R, Tang M, Kohno T, Angulo B, Pio R, Montuenga LM, Minna JD, Yokota J, Sanchez-Cespedes M. A gene-alteration profile of human lung cancer cell lines. Hum Mutat. 2009 Aug;30(8):1199-206. doi: 10.1002/humu.21028. [2] Leonard D. Goldstein, James Lee, Florian Gnad, Christiaan Klijn, Annalisa Schaub, Jens Reeder, Anneleen Daemen, Corey E. Bakalarski, Thomas Holcomb, David S. Shames, Ryan J. Hartmaier, Juliann Chmielecki, Somasekar Seshagiri, Robert Gentleman, David Stokoe, Recurrent Loss of NFE2L2 Exon 2 Is a Mechanism for Nrf2 Pathway Activation in Human Cancers, Cell Reports, 2016, 16 (10): 2605-2617. doi: 10.1016/j.celrep.2016.08.010. From **ATCC**, **Expasy Cellosaurus Database** and **A Gene-Alteration Profile of Human Lung Cancer Cell Lines** *Blanco et al. Hum Mutat 2009.* From NGS (routine diagnostic panel) performed in laboratory (PC435)

**Supplementary Figure 1**

**
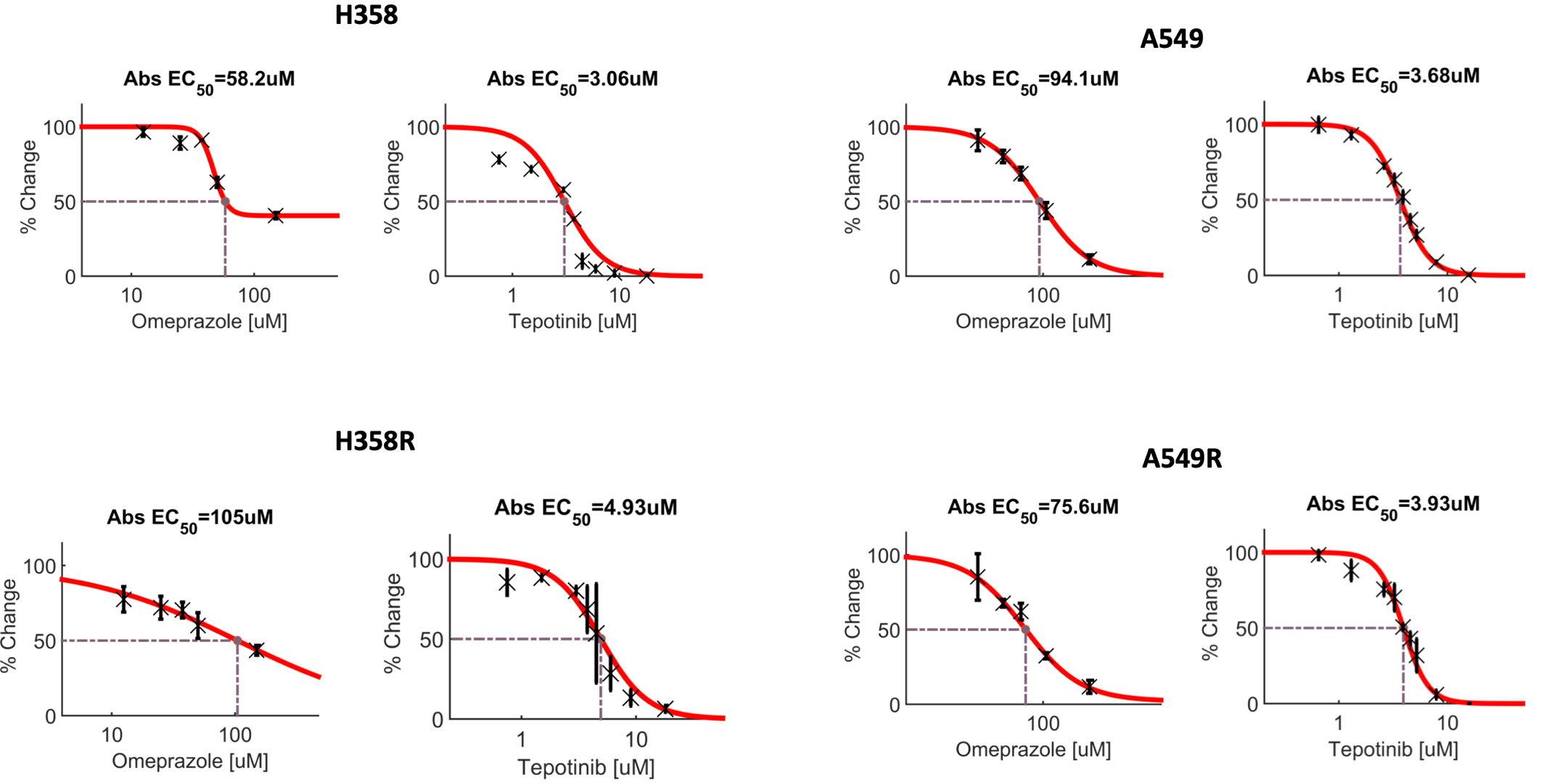
**

**Supplementary Figure 2**

**
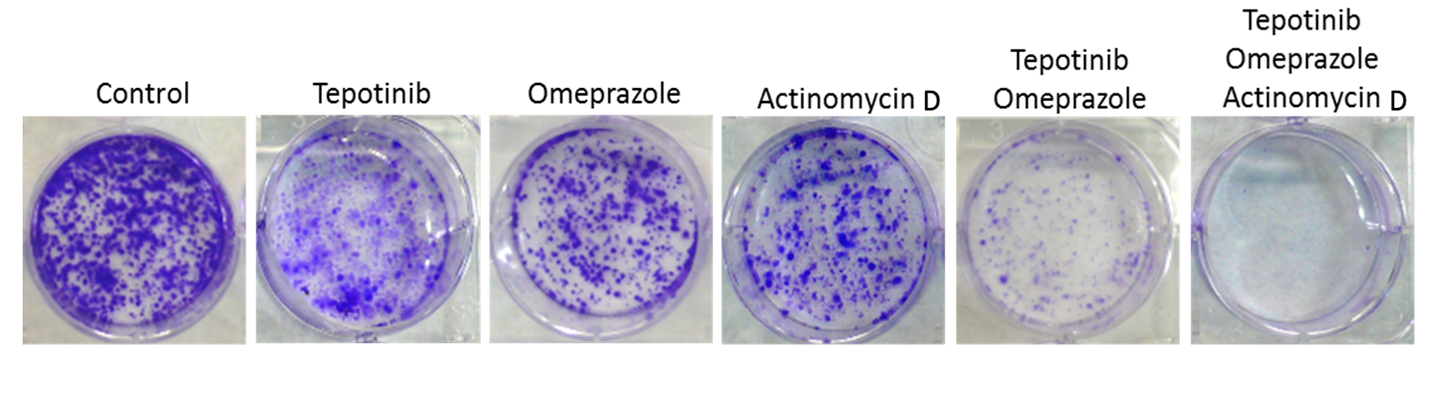
**

**Supplementary Figure 3**

**
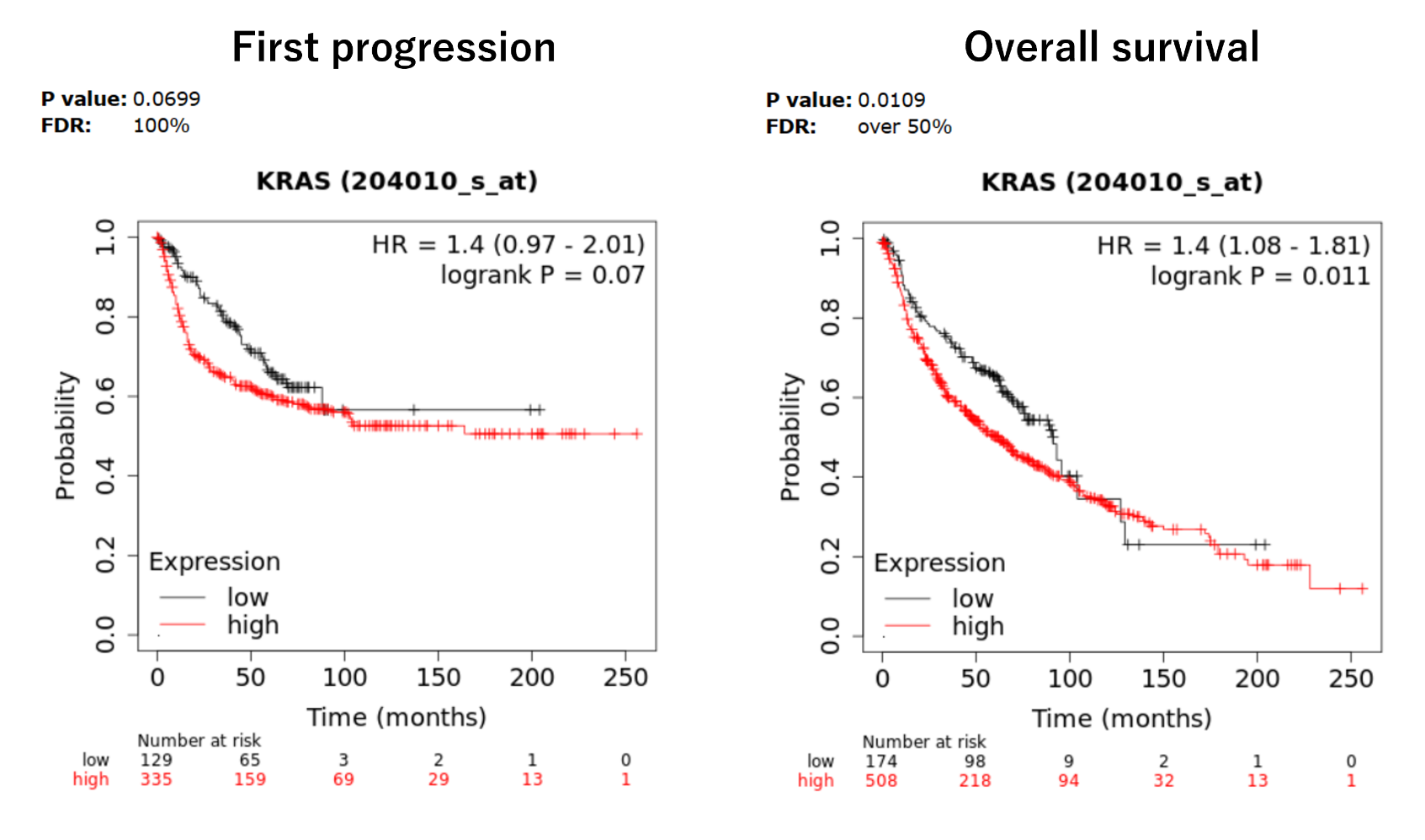
**
